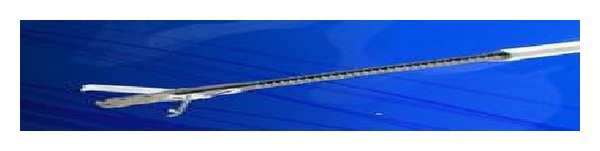# Clinical Application and Outcomes of Over the Scope Clip Device: Initial US Experience in Humans

**DOI:** 10.1155/2013/381873

**Published:** 2013-07-14

**Authors:** Vijay Jayaraman, Christoper Hammerle, Simon K. Lo, Laith Jamil, Kapil Gupta

**Affiliations:** Cedars Sinai Medical Center, Los Angeles, CA 90048, USA

## Abstract

*Background*. OTSCs are now available in the US for various indications. *Methods*. Retrospective review of OTSCs used from January 2011 to April 2012. *Results*. Twenty-four patients underwent placement of 28 OTSCs. Indications included postsurgical fistula, perforations, anastomotic leak, prophylactic closure after EMR, postpolypectomy bleeding, tracheoesophageal fistula, and jejunostomy site leak. Instruments used to grasp the tissue were dedicated (bidirectional forceps or tripronged device) and nondedicated devices (rat/alligator forceps or suction). Success was higher with nondedicated devices (12.5% versus 86.5%, *P* = 0.0004). Overall, OTSC was effective in 15/27 procedures. Defect closure was complete in 12/21. Mean followup was 2.9 months (1–8 m). Mean defect size was 10 mm (5–25 mm). A trend towards higher success was noted in defects <10 mm compared to defects >10 mm (90% versus 60%; *P* = 0.36). No difference was noted in closure of fresh (<72 hrs) versus chronic defects (>1 month) (75% versus 67%). There were no complications. *Conclusion*. The OTSC provides a safe alternative to manage fistula, perforation, and bleeding. No significant difference was seen for closure of early fistula or perforations as compared to chronic fistula. Rat-tooth forceps or suction was superior to the dedicated devices.

## 1. Background

Traditionally, surgical therapy has been the mainstay for closure of perorations and fistula in the gastrointestinal (GI) tract. Recently through the scope, clips have also been used for closure of similar defects and also as therapy for bleeding lesion in the GI tract [1]. However, these clips are limited by width of opening between the jaws and also a lower closure force leading to difficulty in tissue apposition. Multiple clips may therefore need to be placed to close larger defects and for bleeding lesions [2]. Recently an over the scope closure (OTSC—Ovesco Endoscopy, Tuebingen, Germany) device has been made available in the United States for various indications including defect closure and hemostasis. This is a nitinol based clip that is fitted over the scope with a cap. On deployment, it is capable of capturing significantly larger amounts of tissue with more force exerted for tissue apposition. We report here our data regarding the experience with the use of this OTSC device.

## 2. Technique and OTSC Device 

The OTSC is a clip made of shape-memory nitinol alloy. The advantage of nitinol is that the open clip returns to its initial closed shape when it is released from the applicator, causing closure of the two jaws of the clip. It is loaded onto the scope very similar to the band ligator device. Initially a standard endoscopy is performed to identify the defect or lesion, which is to be treated. Depending on the size and appearance of the lesion, an appropriate OTSC is chosen. The OTSC comes in different sizes which can go on different caliber endoscopes. The jaws of the OTSC also vary from blunt (type “a”) for fresh perforations to sharper (types “t” and “gc”) for more fibrotic and chronic defects (Figure 2). The clip is mounted onto a silicone cap (similar to a band ligation device), placed onto the tip of an endoscope, and applied by stretching a wire by means of a hand-wheel installed on the entrance of the working channel. When the clip was released from the applicator, it closes because of the “shape-memory” effect and the high elasticity of the nitinol alloy. This is similar to a bear-claw closure mechanism and applies a permanent force to the tissues. To facilitate targeting the lesion, the kit includes a grasper-shaped anchor with retractable hooks (Figure 3). Additionally there is a twin grasper which has two independently movable jaws that are used to approximate the tissue margins before the OTSC clip is released (Figure 4). Other accessories that were used and are option for tissue apposition included rat tooth and alligator rat tooth forceps. In some small defects, tissue can also be suctioned into the cap and the OTSC device placed. If there was a residual defect that was not closed additional treatment with through the scope clips or stents can also be performed. 

## 3. Patient and Methods

 All patients with attempt at OTSC placement at endoscopy were reviewed at a single tertiary care referral center. Data was collected over a period of one year from January 2011 to April 2012. Procedural data including indication, location of pathology, size of defect, size and number of OTSC used, scope type, accessories used, prior intervention if any, and clinical and technical success rates as well as complications and rates of reintervention and surgery were reviewed. The duration of followup after procedure was also recorded. Patients were followed up by clinical monitoring, drain output, any evidence of recurrent bleeding, and/or radiologic studies. 

## 4. Results

**Figure 1:**
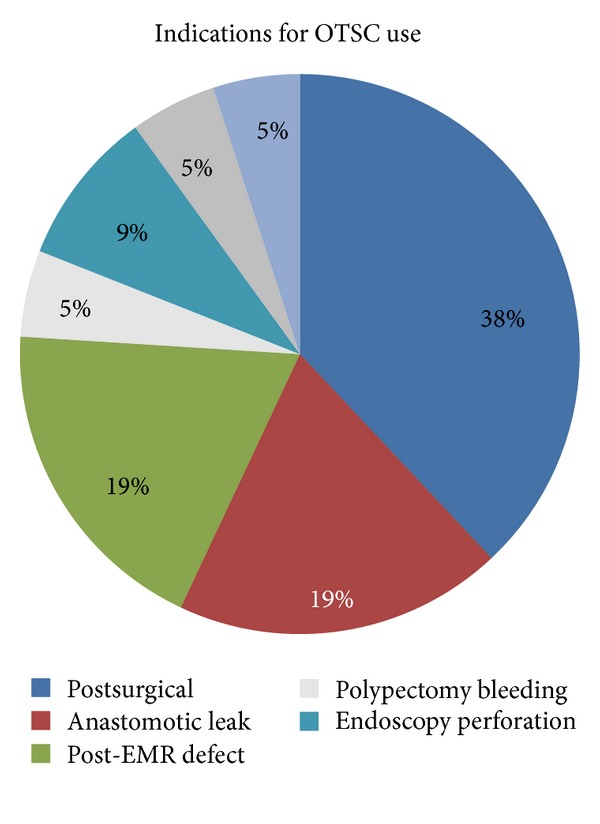


**Figure 2:**
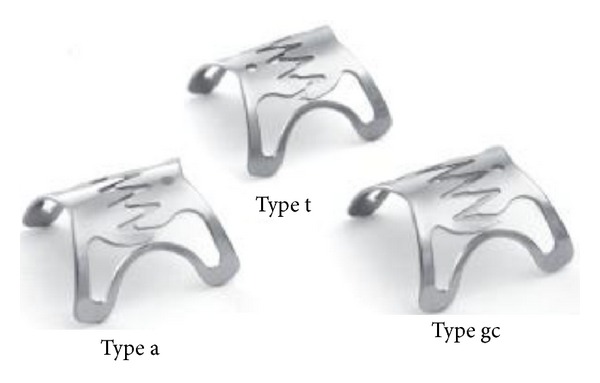


**Figure 3:**
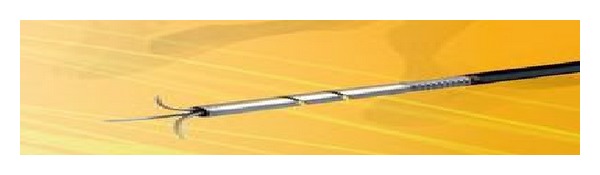


A total of 24 patients underwent placement of 28 OTSC devices. The mean age was 70 years. A total of 14 (58%) patients were female. Indications for OTSC applications (Figure 1) included postsurgical enterocutaneous fistula (10), spontaneous perforation (1), anastomotic leak (4), perforation after mucosal resection (3), prophylactic closure of mucosal defect after EMR (1), postpolypectomy bleeding (2), postendoscopy perforation (2), tracheoesophageal fistula (1), and leakage from a percutaneous jejunostomy site (1). Instruments or modalities used to grasp the tissue were dedicated devices (bidirectional forceps or tripronged anchoring device) in 16 and nondedicated devices (rat tooth/alligator forceps or suction alone) in 15. Successful tissue acquisition with these devices was 2/16 for dedicated and 13/15 for the nondedicated. The nondedicated devices were superior to the dedicated devices for tissue acquisition (*P* = 0.0004). OTSC was successful in achieving hemostasis on a bleeding stump of a large lipoma and a hyperplastic polyp after polypectomy. It was also successful in preventing a post EMR crater from the high risk of delayed bleeding or perforation. Overall, the OTSC was effective in 15/27 procedures (53%). It was used in 21 procedures for defect closure resulting in instant and complete closure in 12/21 (57.1%) and partial closure in 9/21 (42.9%). Mean followup was 2.9 months (range 1–8 m). Mean size of defect was 10 mm (range 5–25 mm). A trend towards higher success was noted in defects <10 mm compared to defects >10 mm (90% versus 60%; *P* = 0.36). The duration of the defect ranged from <24 h to 2 y. No difference was noted in closure of fresh perforations or fistula (<72 hrs) versus chronic fistula (>1 month) (75% versus 67%). Repeat OTSC placement failed to close the defects in 2/2. There were no complications from OTSC device placement. 

## 5. Discussion

Surgery or conservative therapy has been the mainstay of therapy for enteral perforation, anastomotic dehiscence, or fistulae of the gastrointestinal tract which can be associated with mortality rate of up to 5% to 8% [3, 4]. Endoscopic therapy with through the scope clip placement, stents and various sealants has been tried with suboptimal results. Through the scope clips are limited by their smaller wing span and low force of closure necessitating placement of multiple clips for closure of large defects [5]. Stent placements can be limited by wound dehiscence in some cases and migration in 33–80% of cases [6]. Despite these limitations, endoscopic therapy if possible is still the initial choice before any surgical intervention. 

OTSC devices can be used to close large defects [7]. In our series, the overall success rate was 61% with 56% having immediate success and 5% requiring a second endoscopic closure for completion. Surace et al. have reported a similar success rate of 74% in patients for treatment of complications after bariatric surgery [8]. In their subset analysis, they used the OTSC for treatment of complications after sleeve gastrectomy with success in 11/19 patients. A total of 25% of our patients required a repeat procedure which is quite similar to that reported in the literature [8]. Previous series have reported a success rates ranging from 72% [9] to 100% [10]. This might reflect the chronicity of the fistulae in our series (9 out of 24 were chronic, that is, >1 month duration). It has been our experience that success rate of closure of a recent defect is higher compared to chronic fistula although the difference was not statistically different which is likely because of a smaller size of the patients studied. A case can be made to consider endoscopic suturing as first line of therapy in these cases [9]. Also in the series by Kirschniak et al. [10] 7 out of the 11 patients had OTSCs deployed for hemostasis as compared to only 2 in our series and these cases are possibly more amenable to successful treatment than chronic fistulae. Use of OTSCs for hemostasis has been demonstrated in numerous ex vivo models as well [11]. Our case series reports a spectrum of indications for which placement of OTSCs can be useful. 

One of the key elements in success of this technique and placement of OTSC is ability to grasp the tissue successfully. Although many factors like underlying inflammation, fibrosis, and friability duration of fistula can play a role, the device used to grasp the tissue is also important. In our series success with the bidirectional grasping forceps or the anchoring device supplied with the OTSC kit (both manufactured by OVESCO) has been less than satisfactory in terms of tissue acquisition. Our success has been better with a rat tooth or alligator rat tooth forceps possibly because of better grasping ability in the case of fibrotic tissues as the teeth in these forceps tend to be sharper than the dedicated devices. The dedicated devices obviously are extremely useful tools and probably would perform just as well in all other situations; however, rather than using multiple devices, we preferred to use the rat tooth or alligator rat tooth forceps as they worked in all situations. As will be quite obvious, it will be easy to close a smaller size defect and similar results were noted in our series with a trend towards higher rates of successful closure of defects less than 10 mm.

This case series is our experience at a single high volume interventional endoscopy center with all the procedures being performed by one of the three advanced endoscopists. Success of OTSC can also be dependent on the technical skills of the endoscopist. This series is further limited by the retrospective nature although the results from this series do provide a possible safe and effective endoscopic alternative. 

## 6. Conclusion

The OTSC device provides a possible safe and effective endoscopic alternative to manage gastrointestinal fistulae, leaks, bleeding, and perforations. Success of OTSC can depend on size of the defect, chronicity of the defect, and the devices used for grasping the tissue. Larger prospective multicenter studies are needed to further evaluate the safety and success of OTSC. 

## Figures and Tables

**Figure 4:**